# Genetic linkage map construction and QTL mapping of seedling height, basal diameter and crown width of *Taxodium* ‘Zhongshanshan 302’ × *T. mucronatum*

**DOI:** 10.1186/s40064-016-2617-3

**Published:** 2016-06-30

**Authors:** Ziyang Wang, Yanli Cheng, Yunlong Yin, Chaoguang Yu, Ying Yang, Qin Shi, Ziyuan Hao, Huogen Li

**Affiliations:** Institute of Botany, Jiangsu Province and Chinese Academy of Sciences, Nanjing, 210014 Jiangsu China; Key Laboratory of Forest Genetics and Gene Engineering of the Ministry of Education, Nanjing Forestry University, Nanjing, 210037 Jiangsu China

**Keywords:** *Taxodium* ‘Zhongshansa’, Linkage map, QTL mapping, Sequence-related amplified polymorphism (SRAP), Simple sequence repeats (SSR)

## Abstract

*Taxodium* is a genus renowned for its fast growth, good form and tolerance of flooding, salt, alkalinity, disease and strong winds. In this study, a genetic linkage map was constructed using sequence-related amplified polymorphism (SRAP) and simple sequence repeat (SSR) markers based on an F_1_ population containing 148 individuals generated from a cross between *T.* ‘Zhongshanshan 302’ and *T. mucronatum*. The map has a total length of 976.5 cM, with a mean distance of 7.0 cM between markers, and contains 34 linkage groups with 179 markers (171 SRAPs and 8 SSRs). Quantitative trait loci (QTLs) affecting growth traits, such as seedling height, basal diameter and crown width, were detected based on the constructed linkage map. Four significant QTLs were identified, three of which, namely qtSH-1 for seedling height, qtBD-1 for basal diameter and qtCW-1 for crown width, were located at 2.659 cM of LG7 with logarithm odds values of 3.72, 3.49 and 3.93, respectively, and explained 24.9, 27.0 and 21.7 % of the total variation of the three grown traits, respectively. Another QTL for crown width (qtCW-2) was detected at 1.0 cM on LG13, with a logarithm of odds value of 3.15, and explained 31.7 % of the total variation of crown width. This is the first report on the construction of a genetic linkage map and QTL analysis in *Taxodium*, laying the groundwork for the construction of a high-density genetic map and QTL mapping in the genus *Taxodium*.

## Background

*Taxodium* is a genus containing three coniferous species, viz. *Taxodium distichum*, *Taxodium ascendens* and *Taxodium mucronatum* (Qi et al. 2014), which are allogamous, wind-pollinated, and diploid with a haploid chromosome number (n) of 11 (2n = 22). *T. distichum* is native to the southeastern United States, from Delaware to Texas, and inland up the Mississippi River to southern Indiana. It is highly resistant to *Cercosporidium* needle blight and tolerant of flooding, salt, alkalinity and strong winds (Creech et al. 2011). *T. mucronatum* is native to Mexico, much of Guatemala, the tip of South Texas and New Mexico. It is more tolerant of salt and alkaline soils, but less tolerant of flooding and *cercosporidium* needle blight (Creech et al. 2011; Zhou et al. 2010). *T*. ‘Zhongshanshan 302’ is a superior clone selected from a controlled cross between *T. distichum* and *T. mucronatum* (Wang et al. 2015). It is well known for its fast growth, good form, and strong adaptability to a wide range of soils and climates (Cheng et al. 2015). It is also relatively pest-free and has a higher tolerance of salt, alkalinity and flooding (Zhou et al. 2010; Qi et al. 2014). Thus, it has great ecological and economic potential (Cheng et al. 2015). *T*. ‘Zhongshanshan 302’ is registered as a Chinese national variety (Zhou et al. 2010; Wang et al. 2015) and has been widely planted in urban areas and wetlands of eastern China (Zhou et al. 2010; Wang et al. 2015; Cheng et al. 2015; Qi et al. 2014).

Linkage maps facilitate not only gene tagging, map-based cloning (Muchero et al. 2015; Yang et al. 2013a, b), comparative genomic studies (Moriguchi et al. 2012), construction of physical maps, the assembly of whole-genomes (Martínez-García et al. 2013; Marone et al. 2012), and understanding of genome structure and evolution (Scotti et al. 2005; Jermstad et al. 2011), but also molecular marker-assisted selection (MAS) (Moriguchi et al. 2012; Jena et al. 2006) and quantitative trait loci (QTLs) mapping (Moriguchi et al. 2012). In recent decades, genetic maps have been constructed for many conifers, such as *Picea abies* (Scotti et al. 2005; Acheré et al. 2004), *Picea mariana* (Kang et al. 2010; Pelgas et al. 2005), *Pinus taeda* (Martínez-García et al. 2013; Eckert et al. 2009; Temesgen et al. 2001), *Pinus radiata* (Moraga-Suazo et al. 2014), *Pinus pinaster* (Lepoittevin et al. 2012; Rittera et al. 2002), *Pinus koraiensis* (Chen et al. 2010a, b), *Pinus elliottii* (Yang et al. 2013a; Shepherd et al. 2003) and *Pinus lambertiana* (Jermstad et al. 2011). Additionally, MAS for QTLs has been reported in nearly all crop species (Moriguchi et al. 2012; Jena et al. 2006; Zhong et al. 2006). To the best of our knowledge, the majority of genetic maps in conifers have been reported in *Pinaceae*. In *Taxodiaceae*, just *Cryptomeria* and *Cunninghamia* have had several unsaturated maps developed, containing few DNA molecular markers. Tong and Shi (2004) constructed two maps for *C. lanceolata* using 94 and 101 AFLP markers. In 2012, Moriguchi et al. (2012) reported a genetic map for *C. japonica,* in which 1261 SNP markers were mapped on 11 LGs. To date, it is the only high-density genetic linkage map that has been constructed predominantly in *Taxodiaceae*.

Compared with traditional tree improvement approaches that involve the selection of superior trees based on growth characteristics, wood properties or biotic and abiotic stress responses during long growth cycles (Nowicka et al. 2013), QTL mapping offers new opportunities for effective tree breeding (Yoshimaru et al. 1998). QTLs associated with phenotypic variability can be identified accurately by molecular markers in a suitable segregating population (Moraga-Suazo et al. 2014), which has important consequences for employing markers in trees at early stages (Lerceteau et al. 2000) and provides huge potential benefits for improving traits that are difficult, expensive and time-consuming to measure (Nowicka et al. 2013). In recent years, QTL studies based on genetic linkage maps have been reported in several tree species (Nowicka et al. 2013; Lerceteau et al. 2000, 2001; Sewell et al. 2002; Pot et al. 2006; Ukrainetz et al. 2008; Pelgas et al. 2011; Wheelerl et al. 2005).

The use of sequence-related amplified polymorphisms (SRAPs) was initially proposed by Li and Quiros (2001). The PCR-based DNA marker system aims to amplify open reading frames with particular primer pairs (Sun et al. 2006; Li and Quiros 2001; Guo et al. 2014), and its polymorphisms originate from the variations in promoter, intron and spacer lengths among individuals and species (Sun et al. 2006; Li and Quiros 2001). SRAP markers have many advantages, including reliability, reproducibility, simplicity, high efficiency, moderate throughput ratios and easy isolation of bands (Yang et al. 2013a; Chen et al. 2010b; Li and Quiros 2001; Guo et al. 2014). Furthermore, it can target functional genes and detect any base changes, insertions or deletions in a sequence (Yang et al. 2013a; Chen et al. 2010b). Therefore, SRAP can be employed in cDNA fingerprinting, genetic map construction, QTL mapping, comparative genetics and genetic diversity assessments (Yang et al. 2013a; Sun et al. 2006). Yu et al. (2009) established and optimized the SRAP-PCR reaction system in *Taxodium* and successfully identified authentic hybrids generated from a cross of *T. distichum* and *T. mucronatum* using 12 polymorphic SRAP combinations, concluding that SRAP markers are useful and efficient molecular markers in *Taxodium*.

Due to its origin, there are two types of SSR microsatellite markers: genomic SSRs (gSSRs) and expressed sequence tag derived SSRs (EST-SSRs) (Wang et al. 2015; Yang et al. 2013b; Poncet et al. 2006). Compared with gSSRs derived from traditional methods involving the construction of genomic DNA libraries, probe hybridization, cloning and sequencing (Hu et al. 2010; Huang et al. 2011), the development of EST-SSRs with the availability of unaccountable ESTs in public databases, and the advent of Next Generation Sequencing systems, has become a convenient and cost-effective option (Wang et al. 2015; Cheng et al. 2015). SSR markers are considered effective and powerful for assessing genetic diversity and quantifying population genetic structures, relatedness and evolution. This is also true for constructing genetic linkage maps and determining QTLs based on their characteristics of co-dominance, abundance, wide distribution over the genome, and high level of polymorphisms, transferability and reproducibility (Wang et al. 2015; Cheng et al. 2015; Gaudet et al. 2008; Canli 2004; Liu et al. 2014). Thus, microsatellite markers have been widely applied to the construction of genetic linkage maps in forest trees, such as *Eucalyptus grandis* (García et al. 2011), *P. cerasus* (Canli 2004), *P. nigra* (Gaudet et al. 2008), *P. koraiensis* (Chen et al. 2010b), *P. abies* (Acheré et al. 2004), *P. pinaster* (Rittera et al. 2002), *P. elliottii* and *P. caribaea* (Yang et al. 2013a).

This paper reports a first genetic linkage map for *Taxodium* that was constructed using SRAP and SSR markers. Several QTLs associated with seedling height (SH), basal diameter (BD) and crown width (CW) were detected and characterized. The results provide useful information for potential associations between DNA markers and growth traits, and facilitate our understanding of the genome architecture and organization of *Taxodium*.

## Methods

### Plant material and DNA extraction

An F_1_ population comprised of 148 individuals generated from a cross of *Taxodium* ‘Zhongshanshan 302’ and *T. mucronatum* was used as the mapping population. This family was grown in a nursery at the Institute of Botany, Chinese Academy of Sciences in Jiangsu Province (32°02′ N, 118°28′ E; elevation 30 m). *T*. ‘Zhongshanshan 302’ (*T. distichum* × *T. mucronatum*) is a superior clone that was selected in 1988 (Zhou et al. 2010). All of the F_1_ progeny were previously identified as authentic hybrids (Wang et al. 2015). Genomic DNA was extracted from the fresh leaves of each progeny using a modified CTAB method (Wang et al. 2015; Tsumura et al. 1995). The concentration of the extracted DNA was standardized (Moriguchi et al. 2012), and the DNA samples were then stored at –20 °C.

The SH, BD and CW data for each progeny at 4 years of age were measured using diameter tape and Vernier calipers. The location of the BD measurements was on the trunk, 20 cm above the ground. The CW of each seedling was calculated using the mean value of canopy diameter measured along two different orientations: from south to north and from east to west.

Correlation analyses of the SH, BD and CW of the F_1_ population at 4 years of age were performed by SAS 6.12 statistical software (Yao et al. 2016).

### SRAP and EST-SSR profiling

In total, 224 pairs of SRAP primer combinations, created using 14 forward and 16 reverse primers (Table 1; Yang et al. 2013a; Li and Quiros 2001; Yu et al. 2009; Wang et al. 2005), and 503 EST-SSR primer pairs, developed from the transcriptome sequences of *T*. ‘Zhongshanshan 405’ (Wang et al. 2015; Cheng et al. 2015), were used to screen for gene polymorphisms in the parents and four F_1_ hybrid individuals. The polymorphic primer combinations were used in PCR for the mapping population. Loci with null alleles were removed from map construction.

**Table 1:** The forward and reverse primer sequences used in the SRAP-PCR amplification

Forward primer (5′-3′)	Reverse primer (5′-3′)
Me6:TGAGTCCAAACCGGAGA	Em1:GACTGCGTACGAATTAAT
ME7:TGAGTCCAAACCGGACG	Em2:GACTGCGTACGAATTTGC
Me8:TGAGTCCAAACCGGAAA	Em3:GACTGCGTACGAATTGAC
Me9:TGAGTCCAAACCGGAAC	Em4:GACTGCGTACGAATTTGA
Me10:TGAGTCCAAACCGGAAT	Em5:GACTGCGTACGAATTAAC
Me11:TGAGTCCAAACCGGAAG	Em6:GACTGCGTACGAATTCAG
Me12:TGAGTCCAAACCGGTAG	Em7:GACTGCGTACGAATTGAG
Me13:TGAGTCCAAACCGGTTG	Em8:GACTGCGTACGAATTGCC
Me14:TGAGTCCAAACCGGTGT	Em9:GACTGCGTACGAATTTCA
Me15:TGAGTCCAAACCGGTCA	Em10:GACTGCGTACGAATTCAT
Me16:TGAGTCCAAACCGGGAC	Em11:GACTGCGTACGAATTGAT
Me17:TGAGTCCAAACCGGGTA	Em12:GACTGCGTACGAATTCCT
Me18:TGAGTCCAAACCGGGGT	Em13:GACTGCGTACGAATTGCA
Me19:TGAGTCCAAACCGGCAG	Em14:GACTGCGTACGAATTCAA
	Em15:GACTGCGTACGAATTCTG
	Em16:GACTGCGTACGAATTCGA

SRAP-PCR was performed in a total volume of 10 µL containing 50 ng genomic DNA, 1 µL of 10× PCR buffer, 2.0 mmol L^−1^ MgCl_2_, 0.2 mmol L^−1^ dNTPs, 0.3 µmol L^−1^ primers, and 0.5 U Tag DNA polymerase (Shanghai Generay Biotech Co. Ltd, Sanghai, China). PCR reactions were performed in a TC-412 PCR thermal cycler (Bibby Scientific, Stone, United Kingdom) under the following thermal conditions: predenaturation at 94 °C for 4 min; followed by five cycles of denaturation at 94 °C for 1 min, annealing at 37 °C for 1 min, and extension at 72 °C for 1 min; then 35 cycles of denaturation at 94 °C for 1 min, annealing at 50 °C for 1 min and extension at 72 °C for 1 min, followed by a final extension at 72 °C for 7 min.

SSR-PCR amplification was performed in a 10 µL PCR mixture containing 20 ng genomic DNA, 1 µL of 10× PCR buffer, 3.75 mmol L^−1^ MgCl_2_, 0.4 mmol L^−1^ dNTPs, 0.25 µmol L^−1^ primers, and 0.5 U Tag DNA polymerase. The PCR was performed under the following conditions: an initial predenaturation at 94 °C for 3 min, followed by 30 cycles of 30 s at 94 °C, 45 s at the annealing temperature of 59 °C and 45 s at 72 °C, ending with a final extension at 72 °C for 7 min.

The PCR products were stored at 4 °C before being separated on 12 % non-denaturing polyacrylamide gels. Electrophoresis was conducted in 0.5 × TBE buffer (pH 8.0) at 120 V for 1–1.5 h. A 50-bp DNA ladder marker (Takara Biotechnology Co. Ltd, Dalian, China) was used as the molecular standard.

### Genetic linkage map construction

Each band in the electrophoresis gel represented an allelic locus, and the genotypes of individuals from the mapping population could be reconstructed by counting the location and number of bands detected. JoinMap 4.0 was used to construct the linkage map (Van Ooijen 2006). The mapping population in this study could be considered as a cross-pollination population because the genetic background of the two parents was heterozygous. Three segregation type codes <lmxll>, <nnxnp> and <hkxhk> were used to score heterozygous loci in the female parent (*T*. ‘Zhongshanshan 302’), the male parent (*T. mucronatum*), and in both parents, respectively. A Chi square (χ^2^) test was applied to detect whether the inherited alleles of the mapping population were in compliance with the Mendelian segregation ratios. For alleles that were heterozygous in only one of the parents, the segregation ratio across the mapping population was tested against a 1:1 ratio. However, fragments that were heterozygous in both parents were tested against a 3:1 or 1:2:1 ratio. The segregation patterns of markers that did not fit either ratio (P < 0.05) were treated as distorted. Kosambi′s mapping function was used to convert the recombination frequency to a genetic map distance (Kosambi 1944). The “group” command with a logarithm of odds (LOD) threshold of 9.0 and recombination frequency of 0.3 was used to determine all of the linkage groups (LGs). Images of linkage maps were drawn using MapChart 2.1 software (Voorrips 2002).

### Estimation of genome length and map coverage

The observed genome length, G_o_, for the linkage map was calculated as the sum of the sizes of the linkage groups. The expected genome size (G_e_) was estimated using G_e_ = ∑L_i_[(k_i_ + 1)/(k_i_ − 1)] described by Chakravarti et al. (1991), in which L_i_ is the size of the ith LG (cM) and k_i_ is the number of marker loci on the ith LG. Genome coverage was estimated using the ratio between the observed and the expected genome lengths, i.e. G_o_/G_e_.

### QTL analysis

A QTL mapping analysis was performed using interval mapping methods implemented by MapQTL 5.0 (Van Ooijen 2004). A QTL was indicated when the LOD value was higher than the threshold determined using 1000 permutations at a significance level of P = 0.05. The specific location of the QTL was determined using the maximum LOD score in the interval, and confidence intervals (95 %) associated with QTL locations were set as the map intervals corresponding to a 1-point LOD decline on either side of the maximum LOD (Guo et al. 2014). QTLs were named starting with ‘qt’, followed by the abbreviated trait name (SH, BD, or CW) and the number assigned to the QTL.

Female additive (A_f_), male additive (A_m_) and dominant (D) effects of the QTLs were estimated using A_f_ = [(u_ac_ + u_ad_) − (u_bc_ + u_bd_)]/4, A_m_ = [(u_ac_ + u_bc_) − (u_ad_ + u_bd_)]/4, and D = [(u_ac_ + u_bd_) − (u_ad_ + u_bc_)]/4, respectively, where, u_ac_, u_ad_, u_bc_ and u_bd_ are the estimated phenotypic means associated with each of the four possible genotypic classes, ac, bc, ad and bd, respectively, derived from a <abxcd> cross (Guo et al. 2014; Leroy et al. 2011; Qin et al. 2008). The detected QTLs were placed on LGs using the MapChart 2.1 software (Voorrips 2002).

## Results

### Polymorphisms and marker segregation in the mapping population

In total, 113 (50.45 %) of the 224 tested SRAP primer pairs generated 320 polymorphic markers in the F_1_ population. Of the 320 markers, 209 (65.31 %) and 111 (34.69 %) segregated in 1:1 and 3:1 ratios, respectively. Among the 209 markers with a 1:1 ratio, 150 (71.77 %) originated from the female parent and 59 (28.23 %) originated from the male parent (Table 3). The number of polymorphic markers per primer combination ranged from 1 to 5, with an average of 2.83. Among the 320 SRAP segregation markers, 171 (53.44 %) were mapped to the genetic map, which included 97 maternal markers, 7 paternal markers and 67 parental markers.

Furthermore, 257 (51.09 %) out of 503 EST-SSR primer pairs amplified the expected products, of which 17 (3.38 %; Table 2) amplified polymorphic products in the two parents and 4 F_1_ individuals. Of the 17 polymorphic markers, 10 (58.82 %) and 7 (41.18 %) segregated in 1:1 and 1:2:1 ratios, respectively. Among the 10 markers with 1:1 ratios, 9 originated from the female parent and 1 originated from the male parent (Table 3). Among the 17 EST-SSR segregation markers, 8 (47.06 %) were mapped to the genetic map.

**Table 2:** Primers, fragment sizes of EST-SSR

Primer name	Forward primer (5′-3′)	Reverse primer (5′-3′)	Fragment size/bp
TA0106	ATCGTCATCGTCATCGTCCG	TTGTTGAACCGGTGCTGGAT	218
TA0158	GTGCCGGTTTGGGAAATCAC	ACCCCAAATCCACCTGCAAA	252
TA0178	GCCTTTTCTTCTCCCACCCT	CACACCACCCACATTTGCAG	214
TA0197	GGTCAGGGGTTCGATTCTGG	CCACGTTAGCAGGGTTCGAA	277
TA0208	TCTCACTGGTCGAAAGCCAC	CAGAAGGGCCCAAATTCCGA	173
TA0210	GCTTGGAGGTGTTCGAGGAA	GACCCCAGAGTGACAGTTGG	274
TA0214	AGGGGATTGGAAGGAGACGA	ACGTATGTCACCATCCGCTG	240
TA0231	GGTGTTGGAGGAAGGCAAGA	TAATGCCAGATGGTGCTGCA	188
TA0236	TCTTCTTCACCTACCCCCGT	ACCGCCAAAATATCACCCGT	251
TA0283	CAAGCAGAGTCCAAGCCAGA	TGCCTTCACCATGGCCTTAC	210
TA0310	CAGCGGATCCTCTCGATGAC	ATCTAACCGGCAAACCTGGG	230
TA0400	GTAAAGGATTGAGCGCAGTGG	ATGAAGCGCTCTTCCTCTGG	262
TA0430	GCAAGTTACGCCGAGCTTTC	AGGCCCGTTTAATGCAGAGG	109
TA0438	CCGTTGTCTGCACGCAAAAT	AATACCGGAAACCGCTCTGG	229
TA0440	GCCGTACCCTTTTCAGCTCT	CCATGCCGAGACTTACCAGG	268
TA0443	AGATTGCTGGTACCTTGGGC	ATTGGGCCCTCAGGATCTGA	181
TA0448	CCATGGCAGGCGCAAATATC	TTTTGGATTCAACGCTGCCG	134

**Table 3:** Polymorphisms and segregation of the markers in the F_1_ population using JoinMap 4.0

Segregation type	Segregation ratio								No. of markers^a^	No. of distorted markers^a^
	Ll	lm	nn	np	h-	kk	hk	hh		
Lmxll	1	1							150 (9)	18 (3)
nnxnp			1	1					59 (1)	3 (0)
hkxhk					3	1			111 (0)	29 (0)
hkxhk						1	2	1	0 (7)	0 (0)
Total									320 (17)	50 (3)

^a^The numbers indicate the number of sequence-related amplified polymorphism (SRAP) markers and the numbers in parentheses indicate the number of simple sequence repeats (SSR) markers

Among the 337 polymorphic markers (320 SRAPs and 17 EST-SSRs), 284 (84.75 %) showed a normal Mendelian segregation, of which 185 (65.14 %) segregated at 1:1, 82 (28.87 %) segregated at 3:1, and 7 (2.46 %) segregated at 1:2:1 ratios. Additionally, 53 (15.73 %; 50 SRAPs and 3 EST-SSRs) out of the 337 markers showed significant distortions (P < 0.05) from the expected Mendelian segregation ratios (Table 3). Segregation distorted markers were included in the final map only if they did not alter the order of the adjacent markers on the LGs. In total, 49 markers with distorted segregation (47 SRAPs and 2 EST-SSRs) were linked to LGs on the genetic linkage map; 12 and 16 were on LG7 and LG9, respectively, and 21 were on the 15 other LGs (Table 4).

**Table 4:** Map length, map density, and segregation distortion among the 34 LGs in the F_1_ population of *Taxodium*

Linkage group	Map length (cM)	No. of loci	Map density	No. of loci with segregation distortion (*P* < 0.05)	No. of inter-locus gaps (>20 cM)
1	80	12	6.7	3	0
2	54	5	10.8	0	0
3	45.4	4	11.4	1	0
4	44.9	4	11.2	2	1
5	44.8	7	6.4	1	0
6	43.1	8	5.4	1	0
7	42.5	24	1.8	12	0
8	41.9	7	6	1	0
9	39.3	34	1.2	16	0
10	38.2	6	6.4	1	0
11	37.8	5	7.6	0	0
12	36.8	4	9.2	0	0
13	35.3	4	8.8	0	0
14	31.4	4	7.9	0	0
15	30.6	5	6.1	2	0
16	30.2	2	15.1	0	1
17	27.9	3	9.3	0	0
18	26	4	6.5	2	0
19	25.4	2	12.7	0	1
20	25.1	3	8.4	1	1
21	24.4	2	12.2	0	1
22	23	2	11.5	0	1
23	20.1	4	5	0	0
24	17.4	4	4.4	0	0
25	17.1	2	8.6	0	0
26	16.2	2	8.1	0	0
27	14.3	2	7.2	1	0
28	12.9	2	6.5	1	0
29	12.9	2	6.5	0	0
30	11.9	2	6	1	0
31	11	2	5.5	2	0
32	8.3	2	4.2	0	0
33	3.4	2	1.7	0	0
34	3	2	1.5	1	0
Total/mean	976.5	179	7	49	6

### Linkage map construction

**Fig. 1:**
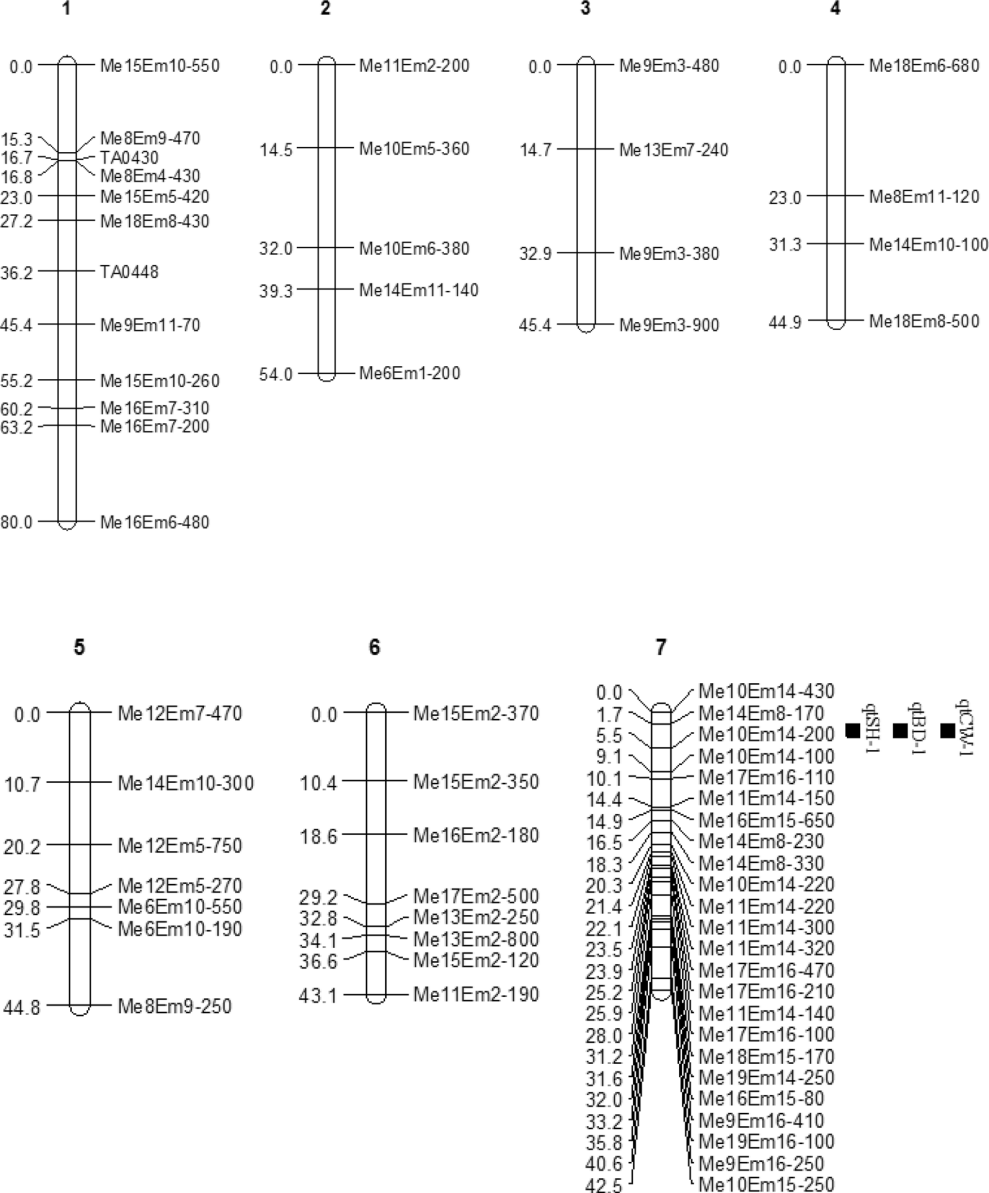
Linkage map from an F_1_ (*T*. ‘Zhongshanshan 302’ × *T. mucronatum*) population, with 171 sequence-related amplified polymorphism (SRAP) and 8 simple sequence repeats (SSRs) markers distributed on 34 linkage groups. The maps had a total length of 976.5 cM. Intervals in cM are shown on the left of each linkage group. The Kosambi function and a logarithm of odds (LOD) threshold of 9.0, and the recombination frequency of 0.3 in JoinMap 4.0 was used to construct the map. MapQTL 5.0 was used to perform quantitative trait locus (QTL) mapping using the interval mapping method. The *bars* along the linkage maps indicate 1-LOD likelihood intervals for the QTLs. The QTLs are for seedling height (qtSH), basal diameter (qtBD), crown width (qtCW) of mapping population

**Fig. 2:**
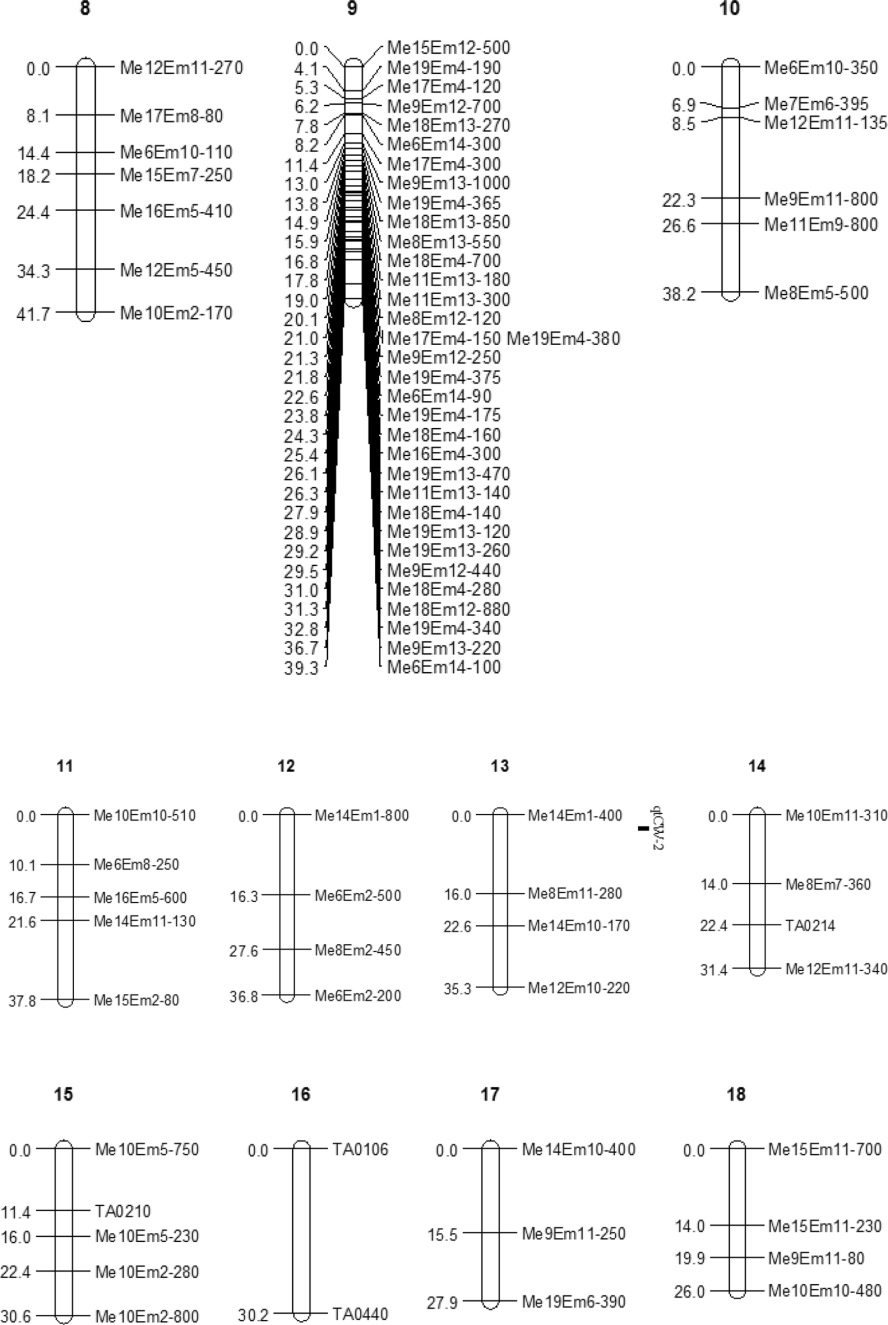
Linkage map from an F_1_ (*T*. ‘Zhongshanshan 302’ × *T. mucronatum*) population, with 171 sequence-related amplified polymorphism (SRAP) and 8 simple sequence repeats (SSRs) markers distributed on 34 linkage groups. The maps had a total length of 976.5 cM. Intervals in cM are shown on the left of each linkage group. The Kosambi function and a logarithm of odds (LOD) threshold of 9.0, and the recombination frequency of 0.3 in JoinMap 4.0 was used to construct the map. MapQTL 5.0 was used to perform quantitative trait locus (QTL) mapping using the interval mapping method. The *bars* along the linkage maps indicate 1-LOD likelihood intervals for the QTLs. The QTLs are for seedling height (qtSH), basal diameter (qtBD), crown width (qtCW) of mapping population

**Fig. 3:**
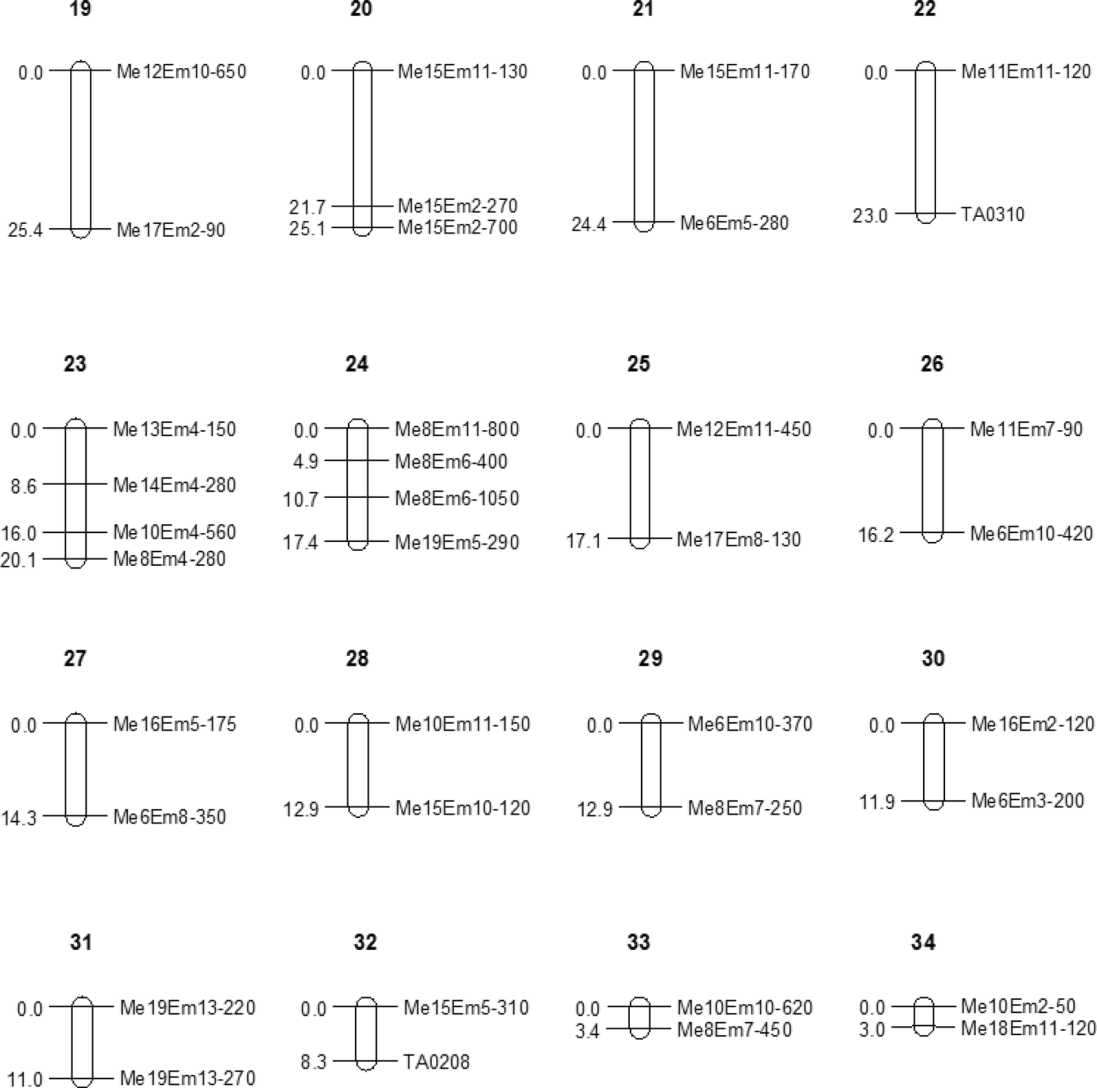
Linkage map from an F_1_ (*T*. ‘Zhongshanshan 302’ × *T. mucronatum*) population, with 171 sequence-related amplified polymorphism (SRAP) and 8 simple sequence repeats (SSRs) markers distributed on 34 linkage groups. The maps had a total length of 976.5 cM. Intervals in cM are shown on the left of each linkage group. The Kosambi function and a logarithm of odds (LOD) threshold of 9.0, and the recombination frequency of 0.3 in JoinMap 4.0 was used to construct the map. MapQTL 5.0 was used to perform quantitative trait locus (QTL) mapping using the interval mapping method. The *bars* along the linkage maps indicate 1-LOD likelihood intervals for the QTLs. The QTLs are for seedling height (qtSH), basal diameter (qtBD), crown width (qtCW) of mapping population

The 179 markers mapped were distributed into 18 groups, plus 2 triples and 14 pairs at the 9.0 LOD threshold. The LGs were named LG1 to LG34 based on their lengths. The 34 LGs contained 2–34 markers, and the map size ranged from 3.0 (LG34) to 80 cM (LG1). All of the seven paternal markers (nnxnp) were associated with maternal markers (lmxll) and bi-parental markers (hkxhk) on LG7 and LG9. The map had a total length of 976.5 cM, with a mean distance of 7.0 cM between markers. The gaps between markers ranged from 0.1 to 30.2 cM. The longest gap (30.2 cM) was between TA0106 and TA0440 on LG16, and gaps longer than 20 cM were located on LG2, LG4, LG16, LG19, LG20, LG21 and LG24 (Figs. 1, 2, 3). The expected genome length of *Taxodium* was 1767.35 cM estimated using method 4 of Chakravarti et al. (1991). The 976.5 cM total size of the linkage map spanned 55.25 % of the estimated *Taxodium* genome length.

### QTL mapping

The results of correlations of the three growth traits of SH, BD and CW showed that there were highly significant (correlations close to 1) among the three traits (Table 5).

**Table 5:** Correlation analysis of seedling height, basal diameter and crown width of F_1_ population

	Seedling height	Basal diameter	Crown width
Seedling height	1.000	0.895	0.901
Basal diameter	0.895	1.000	0.911
Crown width	0.901	0.911	1.000

Three significant QTLs, including one for SH, qtSH-1, one for BD, qtBD-1, and one for CW, qtCW-1, were detected and allocated to the same position at 2.695 cM on LG7, with LOD values of 3.72, 3.49 and 3.93, respectively, between markers Me14Em8-170 and Me10Em14-200 (1.0 cM from Me14Em8-170 and 2.772 cM from Me10Em14-200). qtSH-1 explained 24.9 % of the total variation of SH, qtBD-1 explained 27.0 % of the total variation of BD and qtCW-1 explained 21.7 % of the total variation of CW. A significant QTL for CW, qtCW-2, was detected on LG13. This QTL explained 31.7 % of the total variation of CW and was located at 1.0 cM on LG13, with a LOD value of 3.15. qtCW-2 was 1.0 cM from Me14Em1-400 and 14.953 cM from Me8Em11-280.

The female and male additive, and the dominant effects of the four QTLs were estimated from an output of the program MapQTL 5.0. The results revealed that both parents had positive effects on the four QTLs, which facilitated the SH, BD and CW of *Taxodium*. The dominance effects were positive for qtSH-1, qtBD-1 and qtCW-1, but negative for qtCW-2.

The positions, nearest markers and percentages of the phenotypic variance explained by the QTL (PVE), additive and dominant values, and the directions of the four QTLs are shown in Table 6 and Figs. 1, 2, 3.

**Table 6:** Characterization of the seedling height (SH), basal diameter (BD) and crown width (CW) QTLs in *Taxodium*

QTL	Linkage group	Position^a^ (cM)	Nearest marker	Confidence interval (cM)	LOD^b^	LOD^c^	PVE^d^ (%)	A_f_	A_m_	D
qtSH-1	7	2.7	Me14Em8-170	1.7–3.7	3.72	3.1	24.9	5.59	3.1	23.61
qtBD-1	7	2.7	Me14Em8-170	1.7–3.7	3.49	3.1	27	2.09	1.3	6.53
qtCW-1	7	2.7	Me14Em8-170	1.7–3.7	3.93	3.1	21.7	1.53	0.43	9.84
qtCW-2	13	1	Me14Em1-400	0.0–9.0	3.15	2.4	31.7	6.51	9.9	−1.55

A_f_ female additive effects, A_m_ male additive effects, D dominance effects

^a^Location of the QTL (quantitative trait locus) peak on the corresponding LG

^b^Maximum LOD (logarithm of odds) score (QTL peak)

^c^Threshold LOD score by permutation test

^d^The percentage of the variance explained by the QTL

## Discussion

### Marker polymorphisms

The genetic map was constructed with 179 markers, of which 150 (46.88 %) of the 320 SRAP markers and 9 (52.94 %) of the 17 EST-SSR markers segregated in the maternal parent (*T*. ‘Zhongshanshan 302’), 59 (18.44 %) SRAPs and 1 (5.88 %) EST-SSR segregated in the paternal parent (*T. mucronatum*) and 111 (34.69 %) SRAPs and 7 (41.18 %) EST-SSR segregated in both parents. This difference can be explained by the hybrid origin of *T*. ‘Zhongshanshan 302’ (Wang et al. 2015).

Previously, SRAP technology was successfully applied to analyze genotypes, authenticate hybrid identifications in *Taxodium* (Yu et al. 2009) and to assess the genetic diversity and relationships among 18 *T. mucronatum* individuals of different origins (Zhou et al. 2012). Thus SRAP markers are useful and efficient in *Taxodium*. According to Guo et al. (2014), Yang et al. (2013a), and Chen et al. (2010a), SRAP technology is an efficient method for constructing genetic maps. In this study, we found that the SRAP markers detected highly polymorphic sites in the *Taxodium* genome. A total of 320 polymorphic loci were generated by 113 primer combinations, with a mean of 2.83 polymorphic loci per primer combination. The polymorphic locus ratio of SRAP markers was approximately equal to those of *P. koraiensis* (Chen et al. 2010a) and Zoysiagrass (Guo et al. 2014), but relatively lower than those of other plant species, such as *P. elliottii* (Yang et al. 2013a), *Gossypium hirsutum* (Zhang et al. 2009) and *Saccharum* (Alwala et al. 2008).

Compared with SRAPs, the number of polymorphic SSR markers was insufficient to contribute significantly to the saturation of the map. A total of 17 (3.38 %) SSRs of 503 amplified polymorphic bands in the F_1_ population revealed polymorphisms. The polymorphic ratio was less than that previously reported in other plants such as 12.24 % of pines (Yang et al. 2013a) and 14.4 % of *P. koraiensis* (Chen et al. 2010b). However, due to their specificity and co-dominance, SSR markers were very useful for integrating the parental maps (Acheré et al. 2004). In 2014, Wang et al. (2015) detected the cross-family transferability of 60 primer pairs out of 503 EST-SSRs (including 15 polymorphic primer pairs, 15 primer pairs without polymorphisms and 30 primer pairs without products in *Taxodium*). The results revealed that these primers showed potential cross-family transferability and could be applied to other conifers such as *Taxodiaceae*, *Cupressaceae*, *Pinaceae* and *Taxaceae* (Wang et al. 2015). Therefore, the potential utility of using these microsatellites in comparison and integration of genetic linkage maps in conifer species will undoubtedly increase in the near future.

### Segregation distortion

Segregation distortion is a common phenomenon in the construction of genetic linkage maps (Yang et al. 2013a; Shepherd et al. 2003; Rieseberg et al. 2000) and has been reported in many mapping studies of conifers. Yang et al. (2013a) identified 33.3 and 37.6 % segregation distortion of SRAP, SSR, EST, ISSR markers in *P. elliottii* var. *elliotti* and *P. caribaea* var. *hondurensis*, respectively, similarly the ratios of skewed AFLP markers were ~30 and 35 %, respectively, in the study of Shepherd et al. (2003). Chen et al. (2010a) detected 25.4 % segregation distortions of SRAP, SSR and ISSR in a *P. koraiensis* F_1_ population. Pelgas et al. (2005) detected a 12 % distortion of AFLP, RAPD, SSR and ESTP markers in *P. mariana* × *P. rubens*. Iwata et al. (2001) found that 15 (25 %) out of 60 CAPS markers showed a departure from expected segregation ratios in *Cryptomeria japonica*. He et al. (2000) detected 14.7 % segregation distortion of RAPDs in a *Cunninghamia lanceolata* F_1_ population. Such differences in distorted proportions were likely to have been caused by the variance of the population structure, marker types and genetic mechanisms of each species (Shepherd et al. 2003; Guo et al. 2014).

In this study, 53 (15.73 %) out of 337 markers (320 SRAP and 17 SSR) showed significant segregation distortion, which is comparable to results described above. The skewed markers were distributed on 17 LGs, of which 12 and 16 were on LG7 and LG9, respectively, and 21 were on the 15 other LGs. The results were consistent with other reports in which segregation distortion markers were clustered on several linkage groups (Mukai et al. 1995; Guo et al. 2014; Nodari et al. 1993; Kiss et al. 1993). Even though the underlying mechanism for segregation distortion is still debated, it is recognized that this phenomenon might be due to many complicated factors, including environmental factors, experimental errors and biological factors, such as lethal genes, the presence of fragment-complexes, chromosome loss, viability differences among genotypes, gametic and zygotic selection, non-homologous recombination, and the non-homologous or translocation loci on chromosomes (Mukai et al. 1995; Nikaido et al. 2000; Iwata et al. 2001; Cai et al. 2015).

### Genetic linkage maps

We present here the first report of a genetic linkage map for *Taxodium*. The map spanned 976.5 cM, which covered 55.25 % of the estimated genome length, and was assembled using 179 markers, including 171 SRAP and 8 EST-SSR marker loci arranged on 34 LGs. Compared with other species of *Taxodiaceae*, the map size in our study was shorter than the 1109.1 cM (Iwata et al. 2001), 1405.2 cM (Moriguchi et al. 2012), 1266.1 and 1992.3 cM (Nikaido et al. 2000) of *Cryptomeria japonica* and the 2282.6 and 2565.8 cM (Tong and Shi 2004) of *Cunninghamia lanceolata*. It was larger than *C. japonica* in the study of Mukai et al. (1995), or the 315.3 and 595.2 cM linkage maps of *C. lanceolata* constructed by He et al. (2000). Furthermore, the number of markers mapped was more than in *C. lanceolata* using AFLPs (Tong and Shi 2004), RAPDs (He et al. 2000) and *C. japonica* assembled by CAPS (Nikaido et al. 2000; Iwata et al. 2001), RFLPs, RAPDs (Mukai et al. 1995) and AFLPs (Nikaido et al. 2000). It was less than the map of *C. japonica* constructed using SNPs (Moriguchi et al. 2012). In this study, 179 markers were randomly distributed among 18 groups, 2 triples and 14 pairs. The lengths of these LGs were quite different, ranging from 3 to 80 cM. The discrepancies in marker numbers and map sizes of different groups have been reported in other species such as *P. abies* (Scotti et al. 2005), *P. taeda* (Martínez-García et al. 2013), *C. japonica* (Nikaido et al. 2000), *P. mariana* (Kang et al. 2010), *P. pinaster* (Rittera et al. 2002), *P. sylvestris* (Yin et al. 2003), *P.s nigra* (Gaudet et al. 2008), *P. cerasus* (Canli 2004) and *Triticum turgidum* (Marone et al. 2012). The average distance between the adjacent markers of this map was 7 cM, and six gaps larger than 20 cM were found on six groups. These large gaps may be associated with the lack of more polymorphic markers and a shortage of marker detection in some regions of chromosome (Cai et al. 2015). The genetic linkage maps constructed will provide a foundation for constructing a high density map for *T.* ‘Zhongshansa’ in the future.

There are several defects revealed in this map. On the one hand, in view of the huge genome of *Taxodium*, the map constructed in this paper is only a framework map containing few markers. This defect could be attributed to the closer genetic relationship between the mapping parents and the lower resolution power of marker loci detection means (Wang et al. 2015). Creech et al. (2011), Denny and Arnold (2007) concluded the genus *Taxodium* is a single species with three botanical varieties: baldcypress (*T. distichum* var. *distichum*), pondcypress (*T. distichum* var. *imbricarium*), and montezuma cypress (*T. distichum* var. *mexicanum*). Therefore, the cross of *T*. ‘Zhong shanshan302’ and *T. mucronatum* could be considered as an intraspecific hybridization, and the feeble differences in DNA sequences between the two parents could limit the number of polymorphic markers in the F_1_ population. Moreover, the lower resolution power of the silver-staining detection system may also reduce the number of available segregation markers (Wang et al. 2015). On the other hand, the number of LGs in this map was far more than the haploid chromosome number (n) of 11 (2n = 22) of *Taxodium*. This could be associated with an insufficient quantity of polymorphic markers linked on the map due to the absence of more intermediate loci, leading to gaps that divide chromosomes into several LGs (2 triples and 14 pairs; Guo et al. 2014; Lin et al. 2009). Reports concerning the number of LGs detected are greater than the number of plant chromosomes in several previous studies (Scotti et al. 2005; Komulainen et al. 2003; Shepherd et al. 2003; Iwata et al. 2001; Guo et al. 2014). Scotti et al. (2005) reported a genetic linkage map of *P. abies* (n = 12), which was comprised of 27 LGs containing at least four markers. In 2003, a GL map comprising 27 LGs of *P. elliottii* var. *elliottii*, and a map containing 23 LGs of *P. caribaea* var. *hondurensis* were constructed by Shepherd et al. (2003). These observations might be related to the type and number of individuals of the population, or the type and number of markers used in the mapping (Guo et al. 2014). In addition, LGs of maps typically do not correspond to the haploid chromosome numbers may be because of the nonrandom genomic distribution of different marker types and recombination rates between mapping parents on some chromosomes (Scotti et al. 2005; Cai et al. 2015).

### QTL mapping

The identification of economically important QTLs is a significant foundation for MAS to improve trees and the studies of molecular regulations involved in the various characteristics. The present study represents the first QTL investigation of the growth traits of SH, BD and CW in *Taxodium*. QTLs associated with growth traits have been studied widely in many conifer species (Nowicka et al. 2013; Lepoittevin et al. 2012; Yoshimaru et al. 1998; Ukrainetz et al. 2008; Pelgas et al. 2011; Wheelerl et al. 2005). Ukrainetz et al. (2008) detected two and one QTLs for tree height and diameter at breast height, respectively, in Douglas-fir. Nowicka et al. (2013) successfully searched QTLs related to growth traits of diameter at breast height, of tree height, the number of needles per 10 cm shoots from the apical bud, needle width, needle length and needle area of *P. sylvestris*. Pelgas et al. (2011) identified 137 single QTLs related to growth and phenology, including 33 for bud flush, 52 for bud set and 52 for growth of *Picea glauca*.

Tang et al. (2015) showed that many QTL intervals controlling different fiber quality traits overlapped in some common chromosomal regions. In our study, four major QTLs on LG7 and LG13 were detected (Table 6; Figs. 1, 2, 3), of which one QTL was related significantly to SH (qtSH-1), one to BD (qtBD-1) and one to CW (qtCW-1), which were allocated to the same position at 2.695 cM on LG7, and explained 24.9, 27 and 21.7 % of the total variation of the three growth traits, respectively. The very high correlations among the traits (Table 5), the identical locations of three QTLs and the large phenotypic variances explained suggests that they may be early-growth traits mostly affected by seed size (or amount of storage tissue available to the developing embryo) (Escudero et al. 2000; Sexton et al. 1997). Moreover, these co-localized QTLs may be controlled by pleiotropic genes, which play important roles in the development of growth traits. However, whether the three traits are controlled by the same gene or different genes can still not be determined due to sketchy maps having insufficient markers. Therefore, more markers and a high-density detailed genetic linkage map is needed. The nearest marker of the three QTLs in LG7 was a distorted marker (Me14-Em8-170), and the map distance between this loci and those QTLs was only 1 cM. Previous studies reported that markers having segregation distortion were recognized as potentially powerful evolutionary forces (Cai et al. 2015) and may be associated with several QTLs (Chen et al. 2010a; Xu 2008; Luo et al. 2005).

Changes in biological and climatic factors across years may cause a bias in the phenotypic value assessment of quantitative traits (Chen et al. 2015). In this study, due to the QTLs being detected only in a single year, and with only one individual per genotype of the F_1_ population, there might be instability in this QTL in multiple years and a potential inconsistency in phenotypic assessments between the seedling stage and maturity. Despite addressing these problems, there have been complications in woody plants, and several measures, such as analyzing each of the traits for at least 3 consecutive years and increasing more than three replicates per genotype, are currently underway to ensure that QTLs detected were considered stable and allow us to employ markers in tree breeding accurately and effectively.

## Conclusions

A genetic linkage map was constructed for *T.* ‘Zhongshansa’ using SRAP and EST-SSR markers. A total of 179 markers were distributed to 34 LGs with an observed map length of 976.5 cM and a mean distance of 7.0 cM between markers. In addition, four QTLs related to the growth traits of SH, BD and CW were detected based on the map constructed. Further, it is anticipated that a detailed analysis of QTL locations based on high-density saturated linkage maps of *Taxodium* will be a future task. Additionally, efforts to map more economically important traits, such as growth traits, wood quality and quantitative resistance, which segregate in the *T.* ‘Zhongshansa’ population, are also in progress.
